# On the Use of Iodine in Erysipelas

**Published:** 1841-11-06

**Authors:** Robert Burns

**Affiliations:** Frankford, Pa.


### MEDICAL EXAMINER.

DEVOTED TO MEDICINE, SURGERY, AND THE COLLATERAL SCIENCES.

No. 45.] PHILADELPHIA, SATURDAY, NOVEMBER 6, 1841. [Vol. IV.

### ORIGINAL COMMUNICATION.

### *On the use of Iodine in Erysipelas.*

BY ROBERT BURNS, M. D.

To the Editors of the Medical Examiner.

Gentlemen:—Should you find the following remarks worthy of a corner in your valuable journal, I hope others may be induced, through the same medium, to record their experience of the external use of iodine, and thereby extend to the profession generally the benefits of their observation on this interesting subject.

On the 23d of August, 1841, I was called to visit Mr. William R——d and wife, the former of whom had been sick two weeks previous to my visit, without having any efficient means employed, and his wife several days. On examination, both were found much prostrated, their hands cold and bedewed with perspiration, pulse very feeble, but normal in frequency. Mr. R.'s tongue was of a dark brown colour, chapped over its whole surface, and dry as a piece of bark. The reverse was the case with Mrs. R., her tongue being moist and very little furred, and the only observable departure from its normal condition was slight enlargement, with puffiness of the gums. The faces of both were enormously swelled with erysipelatous inflammation. The colour of the skin was of a dull, dusky red, with several vesicles scattered over the surface. The progress of the inflammation on Mr. R.'s face was in a lateral direction, commencing at the ear; on his wife's it proceeded from below upwards; in both cases the inflammation extended over the whole face, with the exception of the chin, and was passing rapidly upon the hairy scalp when arrested.

Upon the whole, these cases put on a very serious aspect, both as regards their local and general symptoms. In the evening the exacerbation was characterized by excessive heat of the head and face, delirium, protracted insomnia in the case of the female, and frequent starting from sleep in that of the male.

Such a state of things existing, all idea of depletion was at least for the present abandoned, and an alterative treatment, conjoined with diaphoretics and gentle laxatives, seemed to be that which was indicated as best suited to their condition: consequently the following medicines were prescribed on the 23d.

℞. Mass Hydrarg. grs. xii.
Pulv. Rhei, ℈i.
Pulv. Ipecac. comp. grs. vi.
M. ft. pilul. vi.

S. Each to take three at bed time, to be followed by Ol. Risini. in the morning.

℞. Acid Aceti com. ℥ii.

Carb. Ammon. q. s.

Spt. Eth. Nit. ℥ss.

Vin. Ipecac. ℥ii.

Aq. Fontana. ℥ivss.

M. ft. mixture.

S. A table spoonful every hour.

*Aug.* 24.—Bowels freely evacuated with considerable relief. Diaphoretic mixture to be continued; light farinaceous diet; lemonade or Seidlitz powders for drink.

*Aug.* 25.—During the night considerable delirium and wakefulness, which has somewhat subsided.

Continue same diaphoretic, with the addition of ant. tart. gr. iss., instead of the vin. ipecac as above; also one of the following pills every two hours:

℞. Mass. Hydrarg. ℈ii.
Pulv. Ipecac. grs. v.
—— Opii grs. iii.
M. ft. pilul. xx.

*Aug.* 26.—Improved. Mr. R.'s tongue begins to soften at the edges, and its whole surface is more moist. Mrs. R. is also somewhat improved; delirium however continued during the night. Both were ordered to have a blister applied to the nape of the neck, which being done, much relief was produced.

*Aug.* 30.—Convalescence is advancing steadily, under the use of sulph. quininæ and decoct. cinch. comp., varied according to circumstances, and from time the bowels are kept open with infus. sennæ et sulph. magnes.

Having given a rough sketch of the general treatment pursued, we next notice the local measures adopted.

On the evening of the 23d. the ungt. hydrargeri was prescribed, and ordered to be applied spread on strips of linen, about one inch broad, along the line of demarcation. This was continued throughout the first night, and likewise on the 24th. Its application afforded some relief, but produced no further change; the inflammation continued to extend, and I now resolved to use the tincture of iodine, for the knowledge of which, in the treatment of erysipelas, I am, I believe, indebted to the well-stored columns of your useful journal. I was the more favourably impressed with this remedy from having used it about a year ago in erysipelatous inflammation of the foot and knee, and more recently in a case of phlegmon, upon the ala nasi, which produced considerable inflammation and tumefaction of the whole face, and also, in a third instance, upon a highly inflamed and swollen toe; in all of which cases prompt relief, with a speedy termination of the inflammation, was produced. These cases gave me a very favourable impression of the utility of this remedy in local cutaneous inflammation, and the cases which now presented themselves appeared extremely fair ones for a more extensive trial of its utility. Being somewhat timid of using the tincture in its officinal strength, the first application was made dilute, as in the following formula:

℞. Tinct. Iodine f. ʒi.

Spt. V. Rect. f. ʒii.

M. ft. lotio.

With this a portion of the face was penciled over by means of a camel's hair brush. The sensation was to the patient agreeable, and productive of relief in the part to which it was applied; the day following the use of the iodine, the skin was observed to be somewhat wrinkled, and the inflammation partially diminished; the second day (the 26th) the preparation was made stronger, by mixing equal parts of the tincture and spt. v. This was used more extensively than the former; and although the whole inflamed surface, as well as the vesicles both whole and ruptured, were freely varnished over, yet no perceptible bad or even unpleasant effects arose from the medicament; this induced me, on the third day, to employ the tincture without dilution: the effect was now very prompt in destroying the inflammation. On the day following its use, the tumefaction subsided, the epidermis became much corrugated, the effusion in the loose cellular substance of the palpebræ was removed, and in two days after its use desquamation took place, leaving a sound unbroken surface. Even on those places where vesicles had formed, and the fluid was discharged, no sores were produced by the iodine; and, moreover, it was matter of no small satisfaction to observe how desirous these patients were to have their faces painted over at each visit; the sensation, as above remarked, being pleasant, and relieving the disagreeable burning of the inflammation." These patients did well, and on the twelfth day of my attendance were so well as to require no further medical attention. In termino, I have only to say, that, so far as my observation extends, the use of iodine in erysipelas is of extraordinary advantage, and certainly surpasses any other remedy I have ever seen tried. Here, however, the general and local treatment must not be confounded, or the benefits of the former placed to the account of the latter. General treatment in the above cases was absolutely necessary; and this no doubt had a salutary effect upon the local affection: still, it cannot be doubted but the local means was a powerful auxiliary in bringing about a speedy and favourable termination of the disease, and destroying an extensive source of irritation. I will not longer trespass on the patience of your numerous and enlightened readers, but in conclusion hope that some of them will make public their observations on the endermic use of iodine.

*Frankford, Pa.*, Nov. 1, 1841.

